# Integrative taxonomy of a monopisthocotylan (Platyhelminthes, Neodermata) parasite from the gills of *Doras higuchii* Sabaj Pérez & Birindelli (Siluriformes, Doradidae) from the Xingú River Basin, Pará, Brazil

**DOI:** 10.1007/s11230-026-10279-7

**Published:** 2026-05-14

**Authors:** Iasmin L. M. Souza, Geusivam B. Soares, Edson A. Adriano, Marcus V. Domingues

**Affiliations:** 1 Laboratório de Sistemática e Coevolução, Instituto de Estudos Costeiros, Universidade Federal do Pará, Campus Universitário de Bragança, Alameda Leandro Ribeiro, s/n, Bairro Aldeia, Bragança, Pará CEP 68600-000 Brazil; 2 Departamento de Medicina Veterinária, Faculdade de Zootecnia e Engenharia de Alimentos, Universidade de São Paulo, Rua Duque de Caxias, 225, Jardim Elite, Pirassununga, São Paulo CEP 13635-900 Brazil; 3 Instituto de Ciências Ambientais, Quimica e Farmacêuticas, Universidade Federal de São Paulo, Rua Professor Arthur Riedel, 275, Jardim Eldorado, Diadema, São Paulo CEP 09972-270 Brazil; 4 Programa de Pós-Graduação em Biologia Animal, Departamento de Biologia Animal, Instituto de Biologia, Universidade Estadual de Campinas, Rua Monteiro Lobato, 255, Campinas, São Paulo CEP 13083-862 Brazil

## Abstract

This study is an integrative taxonomic analysis of a new species of the genus *Vancleaveus* reported for the first time parasitizing the gills of *Doras higuchii* (Siluriformes, Doradidae) in the Xingú River Basin, Pará, Brazil. *Vancleaveus perditus* **n. sp.** differs from other congeneric species by having a sinistral vaginal aperture, ventral bar with an anteromedian projection, dorsal bar with a posteromedial projection, and a slightly sigmoid MCO with a linguiform distal portion. New partial 28S rDNA sequences were generated for the described species. Using phylogenetic analyses, we found that the new species and *Vancleaveus januacaensis* Kritsky, Thatcher & Boeger, 1986—the type species of the genus—together form a monophyletic lineage. In addition, this study describes the sixth species of *Vancleaveus*.

## Introduction

The Doradidae (Siluriformes), popularly known as talking or thorny catfish, “cuiú-cuiús” or “bacus”, comprises freshwater catfish endemic to South America widely distributed in the tributaries of the Lower Amazon River. They are mainly found in the tributaries, living in the bottom areas of lakes and rivers, while some species are found in in pelagic zones, floodplains, slow streams or river margins (Sabaj & Arce, 2021). Members of this family are characterized mainly by having a line of midlateral row of bony scutes along their sides, which have backward-pointing spines for protection (Birindelli, 2014).

The family comprises 96 valid species in 33 genera (Sabaj & Arce, 2021). Of this diversity, ~75% are recorded for the Amazon basin (Dagosta & De Pinna, 2019) with 15 species specifically documented in the Xingu River (Soares et al., 2018). Among these, *Doras higuchii* Sabaj & Birindelli is widely distributed in the tributaries of the Lower Amazon (Trombetas and Jari rivers) and is the only species of the genus reported from Xingu River (Sabaj Pérez & Birindelli, 2008).

Currently, 29 species of monopisthocotyls parasitizing doradid fish are known, belonging to 4 genera: *Cosmetocleithrum* Kritsky, Thatcher & Boeger, 1986 (24 species), *Pavanelliella* Kritsky & Boeger, 1998 (one species); *Unibarra* Suriano & Incorvaia, 1995 (one species), and *Vancleaveus* Kritsky, Thatcher & Boeger, 1986 (three species) (Table 1). Considering this diversity, five species of monopisthocotyls have been reported from doradids in the Xingu River: *Cosmetocleithrum phryctophallus* Soares, Santos-Neto & Domingues, 2018 and *C. bifurcum* Mendoza-Franco, Mendoza-Palmero & Scholz, 2016 from *Hassar orestis* (Steindachner); *C. leandroi* Soares, Santos-Neto & Domingues, 2019 from *Hassar gabiru* Birindelli, Fayal & Wosiacki; *C. akuanduba* Soares, Santos-Neto & Domingues, 2019 from *H. orestis* and *H. gabiru*; and *Vancleaveus klasseni* Soares, Santos-Neto & Domingues, 2019 from *H. orestis* and *H. gabiru* (Soares et al., 2018). However, there is a lack of information regarding the diversity of monopistocotyls in *Doras higuchii*.

**Table 1:** Diversity of monopisthocotylan parasites of Doradidae

*Cosmetocleithrum amblydorasis* Soares et al. 2026	*Amblydoras affinis*	Soares et al. (2026)
*Cosmetocleithrum akuanduba* Soares, Santos Neto & Domingues, 2018	*Hassar affinis*	Silva et al. (2023)
	*Hassar gabiru*	Soares et al. (2018)
	*Hassar orestis*	Soares et al. (2018)
*Cosmetocleithrum basicomplexum* Silva et al., 2023	*Oxydoras niger*	Silva et al. (2023)
*Cosmetocleithrum bifurcum* Mendoza-Franco, Mendoza-Palmero & Scholz, 2016	*Hassar gabiru*	Soares et al. (2018)
	*Hassar orestis*	Mendoza-Franco et al. (2016), Soares et al. (2018)
*Cosmetocleithrum brachylecis* Silva et al., 2023	*Platydoras brachylecis*	Silva et al. (2023)
*Cosmetocleithrum bulbocirrus* Kritsky, Thatcher & Boeger, 1986	*Pterodoras granulosus*	Kritsky et al. (1986), Silva et al. (2011), Mendoza-Palmero et al. (2012), Pereira et al. (2018), Acosta et al. (2020)
*Cosmetocleithrum confusus* Kritsky, Thatcher & Boeger, 1986	*Oxydoras niger*	Kritsky et al. (1986), Silva et al. (2023)
*Cosmetocleithrum falsunilatum* Feronato, et al., 2022	*Megalodoras uranoscopus*	Feronato et al. (2022), Soares et al. (2026)
*Cosmetocleithrum forcepsiformis* Soares et al. 2026	*Amblydoras affinis*	Soares et al. (2026)
*Cosmetocleithrum gigas* Morey, Cachique & Babilonia, 2019	*Oxydoras niger*	Morey et al. (2020), Soares et al. (2026)
*Cosmetocleithrum gussevi* Kritsky, Thatcher & Boeger, 1986	*Oxydoras niger*	Kritsky et al. (1986), Iannacone & Luque (1991), Silva et al. (2011), Soares et al. (2026)
*Cosmetocleithrum guamaensis* Soares et al. 2026	*Acanthodoras spinossissimus*	Soares et al. (2026)
*Cosmetocleithrum infinitum* Morey, Rojas & Cachique, 2022	*Anadoras grypus*	Morey et al. (2022)
*Cosmetocleithrum leandroi* Soares, Santos Neto & Domingues, 2018	*Hassar affinis*	Silva et al. (2023)
	*Hassar gabiru*	Soares et al. (2018)
*Cosmetocleithrum ludovicense* Silva et al., 2023	*Platydoras brachylecis*	Silva et al. (2023)
*Cosmetocleithrum parvum* Kritsky, Thatcher & Boeger, 1986	*Oxydoras niger*	Kritsky et al. (1986), Silva et al. (2011)
*Cosmetocleithrum phryctophallu*s Soares, Santos Neto & Domingues, 2018	*Hassar orestis*	Soares et al. (2018)
*Cosmetocleithrum rarum* Kritsky, Thatcher & Boeger, 1986	*Oxydoras niger*	Kritsky et al. (1986)
*Cosmetocleithrum sacciforme* Silva, et al., 2023	*Oxydoras niger*	Silva et al. (2023)
*Cosmetocleithrum sobrinus* Kritsky, Thatcher & Boeger, 1986	*Oxydoras niger*	Kritsky et al. (1986), Suriano & Incovaia (1995)
*Cosmetocleithrum taeniophallum* Soares et al. 2026	*Acanthodoras spinossissimus*	Soares et al. (2026)
*Cosmetocleithrum tortum* Mendoza-Franco, Mendoza-Palmero & Scholz, 2016	*Nemadoras hemipeltis*	Mendoza-Franco et al. (2016)
*Cosmetocleithrum undulatum* Silva et al., 2023	*Platydoras brachylecis*	Silva et al. (2023)
*Cosmetocleithrum trachydorasi* (Acosta et al., 2017) Cohen et al., 2020	*Trachydoras paraguayensis*	Acosta et al. (2018), Acosta et al. (2020), Cohen et al. (2020)
*Pavanelliella pavanellii* Kritsky & Boeger, 1998	*Trachydoras paraguayensis*	Acosta et al. (2020)
*Unibarra juruaensis* Justo, Martins & Cohen, 2023	*Oxydoras niger*	Justo et al. (2023)
*Vancleaveus cicinnus* Kritsky, Thatcher & Boeger, 1986	*Franciscodoras marmoratus*	Santos & Brasil-Sato (2006)
*Vancleaveus janauacaensis* Kritsky, Thatcher & Boeger, 1986	*Pterodoras granulosus*	Kritsky et al. (1986), Suriano & Incovaia (1995), Mendoza-Palmero et al. (2012); Pereira et al. (2018),
*Vancleaveus klassen*i Soares, Santos Neto & Domingues, 2018	*Hassar orestis*	Soares et al. (2018), Acosta et al. (2020)
	*Hassar gabiru*	Soares et al. (2018)
***Vancleaveus perditus* Souza, Soares & Domingues n. sp.**	*Doras higuchii*	**Present study**

During an investigation of monopisthocotyls infecting the gills of *Doras higuchii* from the Xingu River Basin, a new species belonging to the genus *Vancleaveus* was found and is described here. In addition, partial 28S rDNA gene sequences were used to analyze the phylogenetic position of the new species in relation to other monopisthocotyls from Neotropical siluriforms.

## Materials and methods

### Host collection

Twenty-five specimens of *Doras higuchii* were collected using a trammel net from the following locations: Gorgulho da Rita, Xingú River, Altamira, Pará State, Brazil (03°21′15.7″S, 52°11′47.5″W); Bacajá River, Altamira, Pará State, Brazil (03°33′47.1″S, 51°36′50.3″W) in February and October of 2015.

### Parasitological procedures

The gill arches were removed and placed in labeled vials containing heated water (~65 °C). Each vial was shaken vigorously, and the sediment and gills were then fixed in 5% formalin for morphological studies or 96% ethanol for molecular characterization. In the laboratory, the content of each vial was examined with a stereoscopic microscope (LEICA S6D, Leica Microsystems, Wetzlar, Germany); the helminths found were removed from the gills or sediments using dissection needles and sent for morphological/ molecular analysis. Specimens intended for studies of internal structures were stained with Gomori’s trichrome (Humason, 1979; Boeger & Vianna, 2006) and mounted in Dammar gum. For the study of sclerotized structures, the specimens were mounted in Hoyer’s or Gray & Wess medium (Humason, 1979; Boeger & Vianna, 2006).

All measurements were made in micrometers following Mizelle & Klucka (1953) and using Leica LAS Interactive Measurement software. Dimensions of organs and other structures represent the greatest measurement in ventral view; lengths of curved or bent structures (anchors, bars, accessory piece) represent the straight-line distances between extreme ends; and the total length of the male copulatory organ (MCO) was measured using the freehand tool in ImageJ software (Rasband, 2022). Hooks were classified according to Mizelle & Price (1963). The average measurement is followed by the ranges and the number (n) of specimens measured in parentheses. Draft illustrations were prepared with the aid of a drawing tube on a microscope with differential interference contrast and phase-contrast optics (LEICA DM 2500, Leica Microsystems, Wetzlar, Germany) using pencil. The final illustrations and plates were prepared in Affinity Designer 2, version 2.6.5. Terminology for plane shapes and outlines in the taxonomic section follows Stearn (1985). The authorship of the new taxa is attributed to the first, second and senior authors following Recommendation 50.1 (Identity of authors) of the International Code of Zoological Nomenclature. Definitions of prevalence and mean intensity were calculated according to Bush et al. (1997). Type specimens, vouchers, and hologenophores (Pleijel et al., 2008) presented in this study were deposited in the following collections: Helminthological Collection of the Instituto Oswaldo Cruz (CHIOC), Rio de Janeiro, Rio de Janeiro State, Brazil; Collection of Platyhelminthes of the Adão José Cardoso, Museum of Zoology of the State University of Campinas (ZUEC PLA), São Paulo State, Brazil. Acting in accordance with the regulations in article 8.5 of the amended 2012 version of the International Code of Zoological Nomenclature, details of the new taxa have been submitted to ZooBank.

### DNA extraction, amplification, and sequencing

The monopisthocotylan specimen used for molecular analysis was initially prepared for genomic DNA extraction as described by Soares et al. (2023). Genomic DNA was extracted using the QIAamp DNA Micro Kit (Qiagen, USA), according to the manufacturer’s protocol, with a final volume of 30 μl. Concentration of the DNA was verified using a NanoDrop One spectrophotometer (Thermo Fisher Scientific, Massachusetts, USA).

The partial 28S rDNA large ribosomal subunit (LSU) nuclear gene was used for genomic amplification, using primer pairs U178 and L1642 (Lockyer et al., 2003). The polymerase chain reaction (PCR) was carried out according to Soares et al. (2023). The PCRs were carried out in a Mastercycler nexus (Eppendorf, Hamburg, Germany) with a final volume of 25 μl and with DreamTaq Green PCR Master Mix (2×) (Thermo Scientific Wilmington, USA), following the manufacturer's recommendations. An amount of 0.1 mM of each primer and 3 μl (~100 ng) of extracted DNA were used in the reactions. To amplify the 28S rDNA fragment, the PCR program was configured for an initial denaturation at 95 °C for 3 min, followed by 34 cycles of 94 °C for 30 s, 56 °C for 30 s, 72 °C for 90 s, and a final elongation at 72 °C for 4 min (Soares et al., 2023). The amplicons were subjected to electrophoresis on a 1.5% agarose gel in TAE buffer (40 mM Tris, 20 mM acetic acid, 1 mM EDTA) stained with SYBRsafe (Invitrogen, Thermo Fisher Scientific, Massachusetts, USA) alongside a 1 kb Plus DNA Ladder (Invitrogen, Thermo Fisher Scientific, Massachusetts, USA) at 100 V for 30 min. The PCR products were purified using the QIAquick PCR Purification Kit (Qiagen, USA) and sequencing was carried out using the BigDye® Terminator Cycle Sequencing Kit v.3.1 Kit (Applied Biosystems™) on an ABI 3730 automatic sequencer (Applied Biosystems, California, USA) from the company OMIKKA (São Paulo State, Brazil), with the same primers used to amplify the 28S rDNA fragment, plus primers 900F, 1200R (Littlewood & Olson, 2001).

### Alignment and Phylogenetic inferences

The contigs generated were edited using Sequencher v.4.1.4 (Gene Codes, Ann Arbor, MI) and subsequently submitted to BLAST analysis (http://blast.ncbi.nlm.nih.gov) to verify similarities with other monopisthocotylan sequences. A total of 47 sequences belonging to the Dactylogyridae published in the NCBI BioSystems database (Geer et al., 2010), along with four sequences of Diplectanidae (*Murraytrema pricei* Bychowsky & Nagibina, 1977, *Pseudorhabdosynochus lantauensis* (Beverley-Burton & Suriano, 1981) and *Pseudorhabdosynochus epinepheli* (Yamaguti, 1938)) and Pseudomurraytrematidae (*Pseudomurraytrema* sp.)(used as outgroups) were retrieved from GenBank, and aligned with the newly generated sequence of *Vancleaveus* from *D. higuchii*. The alignment was generated using default parameters of MUSCLE implemented in Geneious version 7.1.3 (Kearse et al., 2012); the extremes of the alignment were trimmed resulting in 945 bp long. Phylogenetic reconstructions were carried out under Maximum Likelihood (ML) and Bayesian Inference (BI) analyses. The ML analysis was conducted in PhyML v3.0 (Guindon et al., 2010), available through the ATGC–Montpellier web platform, and nodal support was estimated using 1,000 bootstrap (B) replicates. The nucleotide substitution model (GTR + R) was selected based on the Akaike Information Criterion (AIC) implemented in JModelTest v2.1.1. (Darriba et al., 2012).

BI analyses were performed in MrBayes v3.2.6 (Ronquist et al., 2012), under a mixed substitution scheme (lset nst = mixed), incorporating a proportion of invariant sites and gamma-distributed rate variation among sites (rates = invgamma). Posterior probabilities (PP) were inferred from 5 × 10^5^ generations, with two independent runs, each consisting of four simultaneous Markov Chain Monte Carlo (MCMC) chains. Convergence of the Bayesian analyses was confirmed by achieving an average standard deviation of split frequencies below 0.001, and adequate sampling was verified by effective sample size (ESS) values exceeding 200, as assessed in Tracer v1.7 (Rambaut et al., 2018). Trees were sampled at intervals of 1,000 generations, with convergence diagnostics evaluated at the same frequency. The initial 25,000 generations were discarded as burn-in prior to summarizing the posterior distribution. Genetic divergence was determined using the p-distance model matrix in MEGA v7 (Kumar et al., 2016). Trees were visualized using Figtree 1.3.1 (Rambaut, 2012) and figures prepared using CorelDraw 2019.

## Results

We analyzed 25 specimens of *Doras higuchii*, of which 13 (52%) had their gills parasitized by monopisthocotyls. Below, we present the morphological, morphometric, and genetic data from the partial 28S rDNA gene that supports the proposal of a new taxon within the genus *Vancleaveus*.

### Taxonomic section

Class Monopisthocotyla Brabec, Salomaki, Kolisko, Scholz & Kuchta, 2023

Order Dactylogyridea Bychoswhy, 1937

Dactylogyridae Bychoswhy, 1933

*Vancleaveus* Kritsky, Thatcher & Boeger, 1986

### Differential diagnosis

*Vancleaveus* spp. are characterized by lacking eyes; two intestinal caeca, confluent posteriorly to gonads, lacking diverticula; gonads overlapping, testis dorsal to germarium; copulatory complex comprising male copulatory organ (MCO) and accessory piece, non-articulated; MCO sclerotized, tubular, J shaped or slightly sigmoid; vagina singles; vaginal pore dextroventral or sinistroventral, sclerotized; ventral, dorsal anchor pairs present; dorsal anchor with fold on inner superficial root; ventral bar with anteromedial projection present or absent, posteromedial projection present or absent; dorsal bar with anteromedial projection present or absent, posteromedial projection present or absent; hooks (10 ventral, 4 dorsal) with shank inflated.

**Fig. 1:**
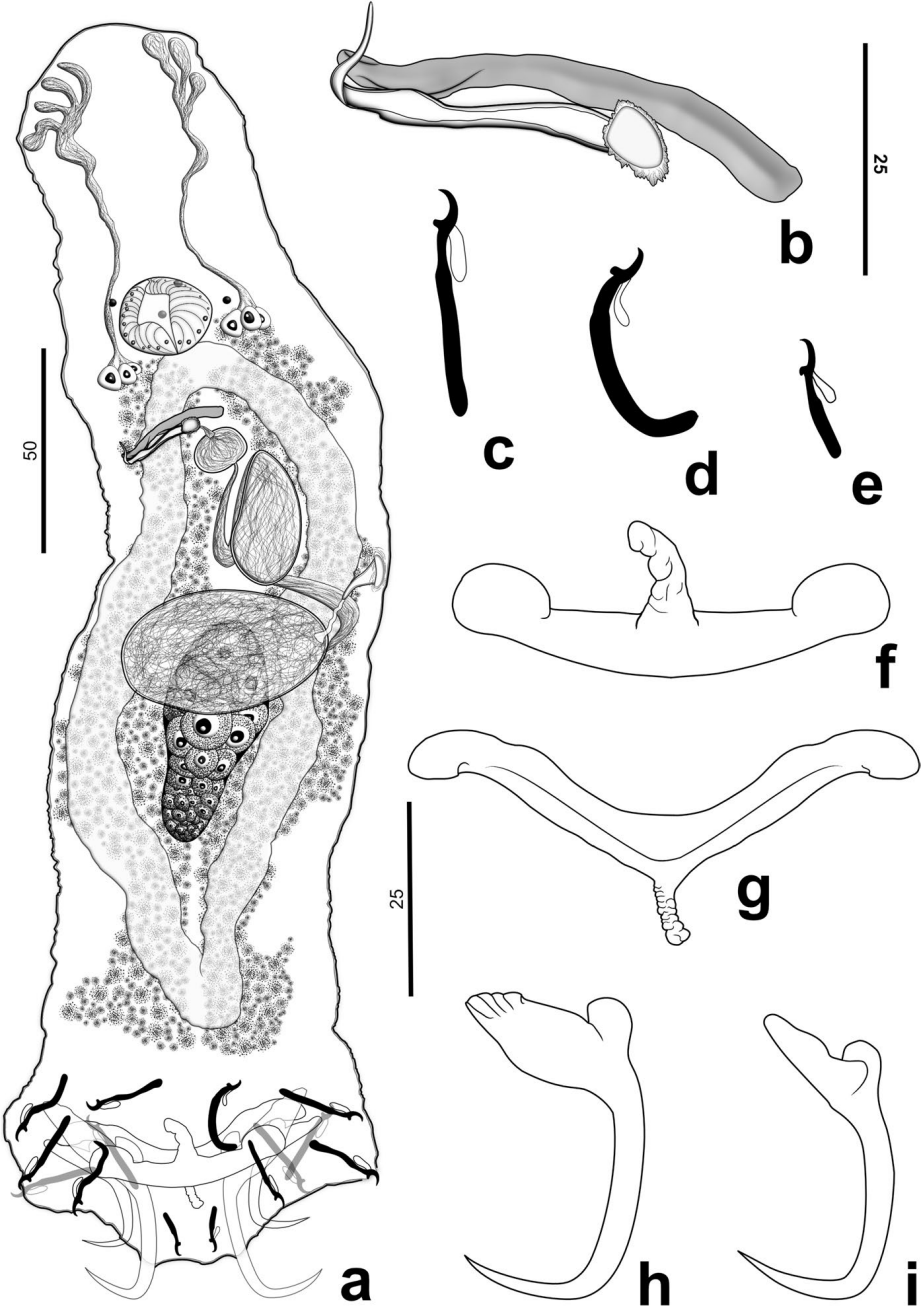
*Vancleaveus perditus* **n. sp.**: a. Whole body (composite), ventral; b. Copulatory complex; c. Hooks II-IV, VI and VII; d. Hook I; e. Hook V; f. Ventral bar; g. Dorsal bar; h. Ventral anchor; i. Dorsal anchor. Figure scales – a: 50 μm scale; b: 25 μm scale; c–i: 30 μm scale

*Vancleaveus perditus* Souza, Soares & Domingues **n. sp.** (Fig. 1)

*Type host*: *Doras higuchii* Sábaj Pérez & Birindelli (Doradidae).

*Type Locality*: Gorgulho da Rita, Xingú River, Altamira, Pará State, Brazil (03°21′15.7″S 52°11′47.5″W).

*Other localities*: Ilha grande, Xingú river, Altamira, Pará State, Brazil (03°35′50.2″S 52°21′22.5″W); Bacajá River, Altamira, Pará State, Brazil (03°33′47.1″S 51°36′50.3″W).

*Site of infestation*: Gill filaments.

*Prevalence*: 52% of 25 hosts examined.

*Average intensity*: 21 parasites per host.

*Average abundance*: 0.5.

*Specimens deposited*: Holotype (CHIOC No. 41038a); 13 paratypes (CHIOC No. 41038b–f, 41039, 41040a–g); 1 voucher (CHIOC No. 41041); 1 hologenophore (ZUEC PLA No. 337).

*Zoobank Life Science Identifier*: C6963939-4A08-4376-AF17-2A1D2528DFCC

*Molecular sequence data*: One 1380 bp long partial sequence of the 28S rDNA gene (accession number: PZ237731).

*Etymology*: The specific name *perditus* (Latin for “lost” or “destroyed”) refers to the type locality where the specimens were collected, which no longer exists due to environmental transformation following the construction of the Belo Monte Dam in the Xingu River, Brazil. The name highlights the loss of the natural environment where this parasite was originally documented.

### Description

Based on 15 specimens, 10 stained in Gomori’s trichrome and 5 mounted in Hoyer' medium. Body elongated, fusiform, 269 (168–410; n = 14) long, excluding haptor, 84 (49–140; n = 14) wide, at level of germarium (Fig. 1a); Tegument smooth. Cephalic region with poor developed cephalic lobes; three to four pairs of head organs present; cephalic glands unicellular, bilateral, posterolateral to pharynx. Chromatic granules present, spherical. Mouth subterminal, midventral; pharynx muscular, globose 23 (30–14; n = 12) in diameter; esophagus short. Genital pore midventral, anterior to copulatory complex. Genital atrium non-sclerotized. Prostatic reservoir, oviduct, Mehlis’ glands, uterus and eggs not observed. Male copulatory organ slightly sigmoid 26 (20–38; n = 8) long**;** base with sclerotized cap, distal portion tapered, linguiform (Fig. 1b). Accessory piece rod-shaped, with grooves at distal portion (Fig. 1b). Testis ovoid 37 (33–41; n = 3) long, 17 (17–18; n = 3) wide; vas deferens looping around left intestinal caecum; seminal vesicle convoluted with proximal portion ovoid, median portion containing descending and ascending loops, transverse broadly elliptical distal portion. Vaginal pore sinistral, ventromarginal; vaginal vestibule non-sclerotized, tulip-like shape; vaginal canal muscular, short, slightly sigmoid; seminal receptacle transverse elliptical. Germarium narrowly obovate 63 (38–84; n = 5) long, 34 (21–53; n = 5) wide. Vitellaria dense, extending from esophagus to confluence of intestinal caecum. Haptor hexagonal 69 (43–122; n = 13) long, 71 (48–115; n = 13) wide. Anchors dissimilar in shape, size. Ventral anchor with well-developed superficial root, broad, inflated at median portion, depressed at distal surface with lengthwise grooves; short deep root, rounded at distal surfaces; shaft elongated, slightly curved at axis; curved point extending to level of tip of superficial root, base 16 (20–13; n = 7), inner 43 (35–48; n = 7), outer 40 (33–44; n = 7) (Fig. 1h). Dorsal anchor with well-developed superficial root, subtriangular, folded at base; short deep root, rounded; shaft elongated, slightly curved at axis; curved point extending past level of tip of superficial root, base 11 (8–13; n = 6), inner 34 (28.5–42; n = 6), outer 34 (28–45; n = 6) (Fig. 1i). Ventral bar 54 (34–68; n = 7) long, 18 (11–23; n = 7) wide, with subtriangular anteromedial projection, terminal portions dilated (Fig. 1f). Dorsal bar 55 (31–66.5; n = 7) long, 13 (10–18; n = 6) wide, open “V” shape, with posteromedial projection, slightly tapered ends, curved in posterior direction (Fig. 1g). Pairs of hooks similar in morphology, shaft with proximal dilation, depressed thumb, slightly recurved shaft, delicate point, delicate hook filament, extending to the limit of shaft dilation. Hook pair I, 30 (26–34; n = 7) long (Fig. 1c); hook pairs II -IV, VI, VII, 32 (26–38; n = 35) long (Fig. 1d); hook pair V, 12 (10–16; n = 7) long (Fig. 1e).

### Remarks

*Vancleaveus perditus* **n. sp.** is distinguished from congeners by a unique combination of features. Firstly, it exhibits a sinistral vaginal aperture, which is also sinistral in *V. klasseni* but dextral in *V. cicinnus* Kritsky, Thatcher & Boeger, 1986*, V. fungulus* Kritsky, Thatcher & Boeger, 1986*, V. janauacaensis* Kritsky, Thatcher & Boeger, 1986, and *V. platyrhynchi* Kritsky, Thatcher & Boeger, 1986. Secondly, the ventral bar of *V. perditus* **n. sp.** lacks a posteromedial projection, a feature that is present exclusively in *V. janauacaensis*. However, the new species possesses an anteromedial projection, which is found in *V. cicinnus*, *V. fungulus*, *V. platyrhynchi*, and *V. klasseni* but is absent in *V. janauacaensis*. Thirdly, the dorsal bar of *V. perditus* **n. sp.** does not have an anteromedial projection, which occurs in *V. cicinnus*, *V. fungulus*, and *V. janauacaensis*, but is absent in *V. klasseni* and *V. platyrhynchi*. Additionally, the dorsal bar of the new species has a posteromedial projection that is absent in all other species. Lastly, *V. perditus* **n. sp.** features a slightly sigmoid MCO in contrast to the J-shaped MCO observed in *V. cicinnus*, *V. fungulus*, and *V. platyrhynchi*, and the coiled form found in *V. janauacaensis* and *V. klasseni*. The new species represents the sixth description of *Vancleaveus* from the Neotropical region.

### Molecular data and phylogenetic inference

**Fig. 2:**
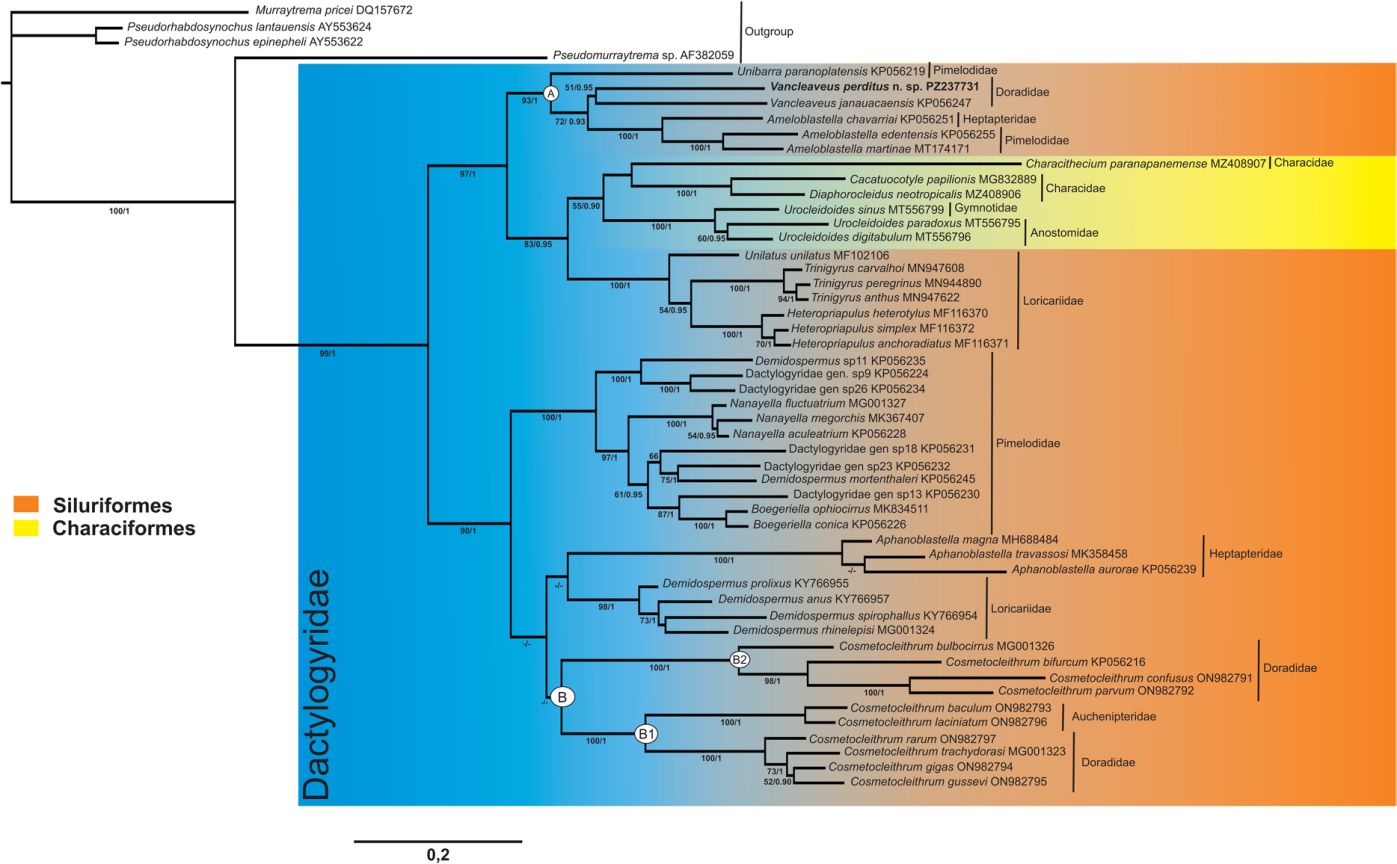
Molecular phylogeny of selected dactylogyrids estimated by maximum likelihood using partial sequences of the 28S rDNA gene. Species sequenced for this study appears in bold. The species name precedes the GenBank sequence ID. ML bootstrap support values and posterior probabilities are given above the branches (bootstrap values < 50 and posterior probabilities <0.90 not reported)

One partial sequence of the 28S rDNA gene were obtained for *Vancleaveus perditus* **n. sp.**– 1380 bp long. The Maximum Likelihood and Bayesian analyses, based on partial 28S rDNA sequences, produced similar tree topology. The ML tree is presented, incorporating statistical support values from both methods (Fig. 2). Most of the clades demonstrated significant statistical support (Fig. 2). Monopisthocotylan species from the Doradidae family arose in two distinct clades. Clade A presents strong support value (B = 93, PP = 1), in which *Vancleaveus perditus* **n. sp.** and *V. janauacaensis* are grouped together with moderate support value (B = 0.95; PP = 51). This clade showed a close relationship with species of *Ameloblastella*, parasites of Pimelodidae and Heptapteridae, with moderate support (B = 72, PP = 0.93). Clade B is divided into two subclades (Clades B1 and B2) both with strong support (B = 100, PP= 1). Clade B1 is formed by *Cosmetocleithrum* spp. from Doradidae (*C. gussevi* Kritsky, Thatcher & Boeger, 1986*, C. gigas* Morey, Cachique & Babilonia, 2019*, C. trachydorasi* (Acosta et al., 2017)*, C. rarum* Kritsky, Thatcher & Boeger, 1986) and Auchenipteridae (*C. laciniatum* Yamada et al., 2017*, C. baculum* Yamada, Yamada & Silva, 2020). Clade B2, consists of *Cosmetocleithrum* spp. that parasitize Doradidae only (*C. bulbocirrus* Kritsky, Thatcher & Boeger, 1986, *C. parvum* Kritsky, Thatcher & Boeger, 1986*, C. confusus* Kritsky, Thatcher & Boeger, 1986*, C. bifurcum*) (Fig. 2).

The genetic divergence between the sequences of *Vancleaveus perditus* **n. sp.** and *V*. *janauacaensis* was 15.2% (234 bp). In comparison, the genetic divergence between the *Vancleaveus* species and those of the phylogenetically related Dactylogyridae genera (Fig. 2) ranged from 15.1% to 15.5% (235-246 bp) for *Ameloblastella* and from 16.2% to 16.6% (260-262 bp) for *Unibarra* (Table 2).

**Table 2:** Pairwise genetic identities of selected partial 28S rDNA sequences of Dactylogyridae species closely related to *Vancleaveus* species adjusted for gaps and missing data

	1	2	3	4	5	6
**1**. *Unibarra paranoplatensis* KP056219		262	260	258	263	266
**2**. *Vancleaveus janauacaensis* KP056247	16.6		234	251	251	246
**3**. ***Vancleaveus perditus* n. sp. PZ237731**	16.2	15.2		235	235	238
**4.** *Ameloblastella chavarriai* KP056251	16.2	15.5	14.7		197	202
**5.** *Ameloblastella edentensis* KP056255	16.6	15.4	14.9	11.4		104
**6.** *Ameloblastella martinae* MT174171	16.5	15.1	14.9	11.6	6.4	

The upper triangular matrix shows the number of differences in nucleotides while the lower triangular matrix shows the differences in terms of percentage of nucleotides

Sequences obtained in the present study are shown in in boldface type

## Discussion

The proposal for the new species is based on a comprehensive analysis of the morphological and molecular data of members of the *Vancleaveus* spp. lineage. This newly described species is the first dactylogyrid known to parasitize hosts from the genus Doras. The discovery increases the number of monopisthocotylan species associated with Doradidae to thirty.

The genus *Vancleaveus* was proposed by Kritsky et al. (1986) to accommodate four new species: *V. janauacaensis* (type-species) from doradid fish; and *V. cicinnus*, *V. fungulus*, and *V. platyrhynchi* from pimelodids in the Amazon River basin. Subsequently, Santos & Brasil-Sato (2006) reported *V. cicinnus* from *Franciscodoras marmoratus* (Lütken), a doradid from the São Francisco River in Brazil. Twelve years later, Soares et al. (2018) described *V. klasseni* from the gills of *Hassar orestis and H. gabiru* from the Xingu River basin*.*

Members of this genus are characterized mainly by having MCO non-articulated to the accessory piece, ventral vaginal pore, dorsal anchors with conspicuous basal folds on the superficial root, and a ventral bar with a medial projection (Kritsky et al., 1986; Boeger & Vianna, 2006). We observed variations in the position of the medial projection of the ventral bar and the location of the vaginal pore among different species. Specifically, *Vancleaveus janauacaensis*, *V. fungulus*, and *V. platyrhynchi* exhibit a posteromedial projection on the ventral bar, while *V. cicinnus and V. klasseni* have an anteromedial projection. Regarding the vaginal pore, *V. cicinnus*, *V. fungulus*, *V. janauacaensis*, and *V. platyrhynchi* possess a ventromedial vaginal pore, whereas *V. klasseni* features a sinistral, ventromarginal vaginal pore. Here, *Vancleaveus perditus* **n. sp.** is described by possessing a combination of these features (an anteromedial projection in the ventral bar and a sinistral, ventromarginal vaginal pore), as well as unique characteristics like a dorsal bar with a posteromedial projection, and a slightly sigmoid MCO with a linguiform distal portion.

Our phylogenetic analysis (Fig. 2) provides support for *Vancleaveus perditus* **n. sp.** as a valid member of the genus, as it is grouped with *V. janauacaensis* (type-species), which is so far the only available sequence of the genus. These two species exhibited a significant genetic divergence of 15.2% (234 bp). Our results support previous studies (Mendoza-Palmero et al., 2015, 2022), that showed *Vancleaveus* spp. closely related to *Ameloblastella* species infecting Pimelodidae and Heptapteridae, as well as to *Unibarra paranoplatensis,* which also infects Pimelodidae. As Mendoza-Palmero et al. (2015) reported, our findings further show that even monopisthocotylan parasites infecting the same doradid fish species can be phylogenetically distant.

For example, *V. janauacaensis* and *C. bulbocirrus*, both parasites of *Pterodoras granulosus,* are found in separate groups: clades A and B, respectively. Similarly, five *Cosmetocleithrum* species (*C. gussevi*, *C. gigas*, *C. rarum*, *C. parvus*, and *C. confusus*) infecting *Oxidoras niger* are grouped within clades B1 and B2. We also observed that different species of *Vancleaveus* can infect unrelated Neotropical Siluriformes families, such as *Vancleaveus cicinnus*, which have been reported in both Pimelodidae and Doradidae. Mendoza-Palmero et al. (2015) also suggested that independent colonization and host-switching events contribute to the diversification and distribution of parasitic monopisthocotyls across South American siluriforms. Our findings support this view, and we emphasize that future cophylogenetic studies could further clarify how these monopisthocotylan lineages and their siluriform hosts have evolved in South America.

This study advances our knowledge of monopisthocotylan species in Doradidae, particularly in the Amazon basin. By combining morphological and molecular data, we identified a new *Vancleaveus* species, underscoring the value of integrative taxonomy. The research also highlights the need for more studies of monopisthocotylan parasites in Neotropical fish, and advocates using both molecular and morphological data to clarify phylogenetic relationships and coevolution in this host-parasite system.

## Data Availability

No datasets were generated or analysed during the current study.
